# 16-Methyl-11-(2-methyl­phen­yl)-14-phenyl-8,12-dioxa-14,15-di­aza­tetra­cyclo­[8.7.0.0^2,7^.0^13,17^]hepta­deca-2(7),3,5,13(17),15-penta­ene-10-carbo­nitrile

**DOI:** 10.1107/S1600536813029905

**Published:** 2013-11-06

**Authors:** Jeevanandam Kanchanadevi, Gopalakrishnan Anbalagan, Damodharan Kannan, Manickam Bakthadoss, Vadivelu Manivannan

**Affiliations:** a Department of Physics, Velammal Institute of Technology, Panchetty, Chennai 601 204, India; b Department of Physics, Presidency College (Autonomous), Chennai 600 005, India; c Department of Organic Chemistry, University of Madras, Guindy campus, Chennai 600 025, India; d Department of Research and Development, PRIST University, Vallam, Thanjavur 613 403, Tamil Nadu, India

## Abstract

In the title compound, C_28_H_23_N_3_O_2_, the pyrazole ring makes a dihedral angle of 16.90 (6)° with the phenyl ring to which it is attached. Both di­hydro­pyran rings exhibit half-chair conformations. Intramolecular C—H⋯O interactions generate *S*(6) and *S*(8) ring motifs. In the crystal, weak C—H⋯O and C—H⋯π interactions occur.

## Related literature

For the biological activities of 4*H*-chromenes see: Cai *et al.* (2006 ▶); Gabor (1988 ▶); Brooks (1998 ▶); Valenti *et al.* (1993 ▶); Tang *et al.* (2007 ▶). For related structures see: Ponnusamy *et al.* (2013 ▶); Kanchanadevi *et al.* (2013 ▶).
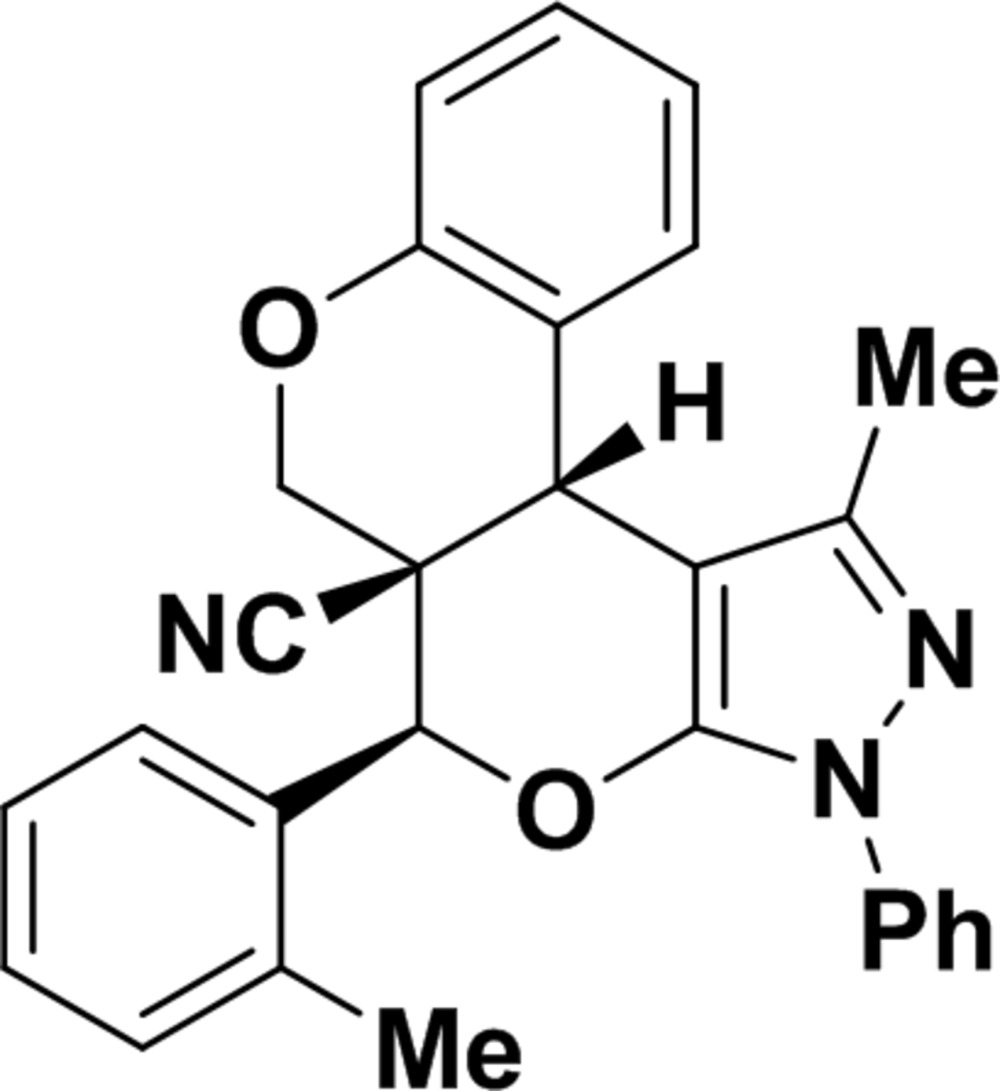


## Experimental

### 

#### Crystal data

C_28_H_23_N_3_O_2_
*M* *_r_* = 433.49
Triclinic, 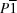

*a* = 9.021 (5) Å
*b* = 9.604 (5) Å
*c* = 15.254 (5) Å
α = 72.720 (5)°
β = 76.997 (5)°
γ = 63.875 (5)°
*V* = 1126.1 (9) Å^3^
*Z* = 2
Mo *K*α radiation
μ = 0.08 mm^−1^
*T* = 295 K
0.30 × 0.20 × 0.20 mm

#### Data collection

Bruker APEXII CCD diffractometer
Absorption correction: multi-scan (*SADABS*; Sheldrick, 1996 ▶) *T*_min_ = 0.980, *T*_max_ = 0.983
20066 measured reflections
4154 independent reflections
3085 reflections with *I* > 2σ(*I*)
*R*_int_ = 0.034

#### Refinement

*R*[*F* ^2^ > 2σ(*F* ^2^)] = 0.043
*wR*(*F* ^2^) = 0.133
*S* = 1.03
4154 reflections
300 parameters
H-atom parameters constrained
Δρ_max_ = 0.41 e Å^−3^
Δρ_min_ = −0.18 e Å^−3^

### 

Data collection: *APEX2* (Bruker, 2008 ▶); cell refinement: *SAINT* (Bruker, 2008 ▶); data reduction: *SAINT*; program(s) used to solve structure: *SHELXS97* (Sheldrick, 2008 ▶); program(s) used to refine structure: *SHELXL97* (Sheldrick, 2008 ▶); molecular graphics: *PLATON* (Spek, 2009 ▶); software used to prepare material for publication: *SHELXL97*.

## Supplementary Material

Crystal structure: contains datablock(s) I. DOI: 10.1107/S1600536813029905/bt6941sup1.cif

Structure factors: contains datablock(s) I. DOI: 10.1107/S1600536813029905/bt6941Isup2.hkl

Supplementary material file. DOI: 10.1107/S1600536813029905/bt6941Isup3.cml

Additional supplementary materials:  crystallographic information; 3D view; checkCIF report

## Figures and Tables

**Table 1:** Hydrogen-bond geometry (Å, °) *Cg*1 is the centroid of the C1–C6 ring.

*D*—H⋯*A*	*D*—H	H⋯*A*	*D*⋯*A*	*D*—H⋯*A*
C6—H6⋯O1	0.93	2.37	2.952 (2)	120
C28—H28*C*⋯O2	0.96	2.47	3.310 (3)	146
C16—H16*A*⋯O2^i^	0.97	2.56	3.380 (2)	143
C20—H20⋯*Cg*1^ii^	0.96	2.86	3.567 (3)	134

Symmetry codes: (i) 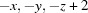; (ii) 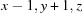.

**Fig. 1.**
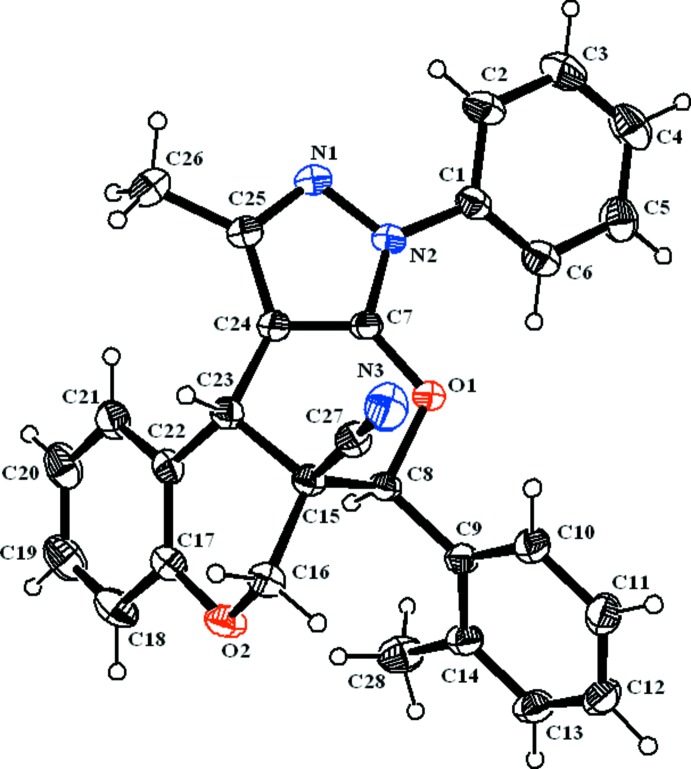
The molecular structure of the title compound, with atom labels and 30% probability displacement ellipsoids for non-H atoms.

**Fig. 2.**
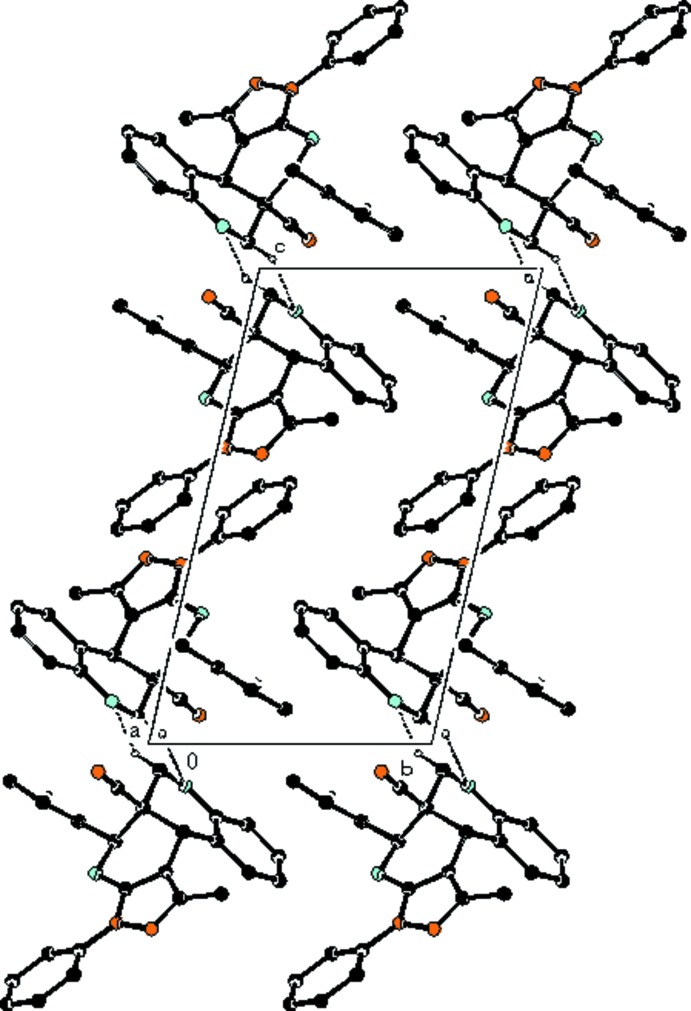
The packing of the title compound, viewed down the a axis. Hydrogen bonds are shown as dashed lines.

## supplementary crystallographic information

### 1. Comment

4*H*-Chromenes and their derivatives exhibit various biological activities such as anti-viral, anti-fungal, anti-inflammatory, antidiabetic, anti-anaphylactic and anti-cancer (Cai *et al.*, 2006; Gabor, 1988; Brooks, 1998; Valenti *et al.*, 1993; Tang *et al.*, 2007).

The geometric parameters of the title molecule (Fig. 1) agree well with reported similar structures (Ponnusamy *et al.*, 2013; Kanchanadevi *et al.*, 2013). The pyrazole ring makes a dihedral angle of 16.90 (6) ° and 69.04 (6) °, respectively with two phenyl [C1—C6 and C9—C14] rings.

The molecular structure is stabilized by weak intramolecular C—H···N, C—H···O interactions and the crystal packing is controlled by weak intermolecular C—H···O and C—H···π interactions [C20—H20···Cg1^ii^; H20···cg1^ii^ 2.863Å, C20—H20···Cg1^ii^ 134°; Cg1 is the centroid of the ring defined by the atoms C1—C6; symmetry operator for generating equivalent atoms: (ii) -1 + *x*, 1 + *y*, *z*].

### 2. Experimental

A mixture of (2E)-3-(2-methylphenyl)-2-(2-formylphenoxymethyl) prop-2-enenitrile (0.28 g, 1 mmol) and 3-methyl-1-phenyl-1*H*-pyrazol -5-one (0.17 g, 1 mmol) was placed in a round bottom flask and melted at 200 ° C for 1 h. After completion of the reaction as indicated by TLC, the crude product was washed with 5 ml of ethyl acetate and hexane mixture (1:49 ratio) which successfully provided the title product as colorless solid in 93% yield.

### 3. Refinement

H atoms were positioned geometrically and refined using a riding model with C—H = 0.93 Å and *U*_iso_(H) = 1.2*U*_eq_(C) for aromatic C—H, C—H = 0.98 Å and *U*_iso_(H) = 1.2*U*_eq_(C) for C—H, C—H = 0.97 Å and *U*_iso_(H) = 1.2*U*_eq_(C) for CH_2_, C—H = 0.96 Å and *U*_iso_(H) = 1.5*U*_eq_(C) for CH_3_. The methyl groups were allowed to rotate but not to tip.

### Crystal data

**Table:**

C_28_H_23_N_3_O_2_	*Z* = 2
*M**_r_* = 433.49	*F*(000) = 456
Triclinic, *P*1	*D*_x_ = 1.278 Mg m^−^^3^
Hall symbol: -P 1	Mo *K*α radiation, λ = 0.71073 Å
*a* = 9.021 (5) Å	Cell parameters from 9100 reflections
*b* = 9.604 (5) Å	θ = 2.4–25.4°
*c* = 15.254 (5) Å	µ = 0.08 mm^−^^1^
α = 72.720 (5)°	*T* = 295 K
β = 76.997 (5)°	Block, colourless
γ = 63.875 (5)°	0.30 × 0.20 × 0.20 mm
*V* = 1126.1 (9) Å^3^	

### Data collection

**Table:**

Bruker APEXII CCD diffractometer	4154 independent reflections
Radiation source: fine-focus sealed tube	3085 reflections with *I* > 2σ(*I*)
Graphite monochromator	*R*_int_ = 0.034
Detector resolution: 0 pixels mm^-1^	θ_max_ = 25.4°, θ_min_ = 2.4°
ω and φ scans	*h* = −10→10
Absorption correction: multi-scan (*SADABS*; Sheldrick, 1996)	*k* = −11→11
*T*_min_ = 0.980, *T*_max_ = 0.983	*l* = −18→18
20066 measured reflections	

### Refinement

**Table:**

Refinement on *F*^2^	Primary atom site location: structure-invariant direct methods
Least-squares matrix: full	Secondary atom site location: difference Fourier map
*R*[*F*^2^ > 2σ(*F*^2^)] = 0.043	Hydrogen site location: inferred from neighbouring sites
*wR*(*F*^2^) = 0.133	H-atom parameters constrained
*S* = 1.03	*w* = 1/[σ^2^(*F*_o_^2^) + (0.0709*P*)^2^ + 0.226*P*] where *P* = (*F*_o_^2^ + 2*F*_c_^2^)/3
4154 reflections	(Δ/σ)_max_ = 0.003
300 parameters	Δρ_max_ = 0.41 e Å^−^^3^
0 restraints	Δρ_min_ = −0.18 e Å^−^^3^

### Fractional atomic coordinates and isotropic or equivalent isotropic displacement parameters (Å^2^)

**Table:**

	*x*	*y*	*z*	*U*_iso_*/*U*_eq_	
C1	0.6925 (2)	−0.0765 (2)	0.56488 (12)	0.0419 (4)	
C2	0.8398 (3)	−0.0745 (3)	0.51420 (15)	0.0625 (6)	
H2	0.8788	−0.0018	0.5180	0.075*	
C3	0.9283 (3)	−0.1800 (3)	0.45841 (16)	0.0732 (7)	
H3	1.0279	−0.1791	0.4250	0.088*	
C4	0.8716 (3)	−0.2860 (3)	0.45144 (15)	0.0688 (6)	
H4	0.9324	−0.3577	0.4138	0.083*	
C5	0.7238 (3)	−0.2861 (3)	0.50042 (14)	0.0614 (6)	
H5	0.6840	−0.3572	0.4951	0.074*	
C6	0.6334 (2)	−0.1812 (2)	0.55777 (13)	0.0491 (5)	
H6	0.5337	−0.1820	0.5910	0.059*	
C7	0.4881 (2)	0.0363 (2)	0.69459 (11)	0.0364 (4)	
C8	0.28622 (19)	−0.0390 (2)	0.79451 (11)	0.0349 (4)	
H8	0.1918	0.0427	0.7618	0.042*	
C9	0.2496 (2)	−0.1847 (2)	0.83666 (11)	0.0363 (4)	
C10	0.3741 (2)	−0.3256 (2)	0.87619 (13)	0.0441 (4)	
H10	0.4801	−0.3293	0.8723	0.053*	
C11	0.3442 (3)	−0.4593 (2)	0.92089 (13)	0.0507 (5)	
H11	0.4293	−0.5527	0.9463	0.061*	
C12	0.1869 (3)	−0.4537 (2)	0.92762 (13)	0.0519 (5)	
H12	0.1640	−0.5421	0.9598	0.062*	
C13	0.0645 (2)	−0.3173 (2)	0.88667 (13)	0.0493 (5)	
H13	−0.0404	−0.3160	0.8903	0.059*	
C14	0.0913 (2)	−0.1809 (2)	0.83990 (12)	0.0415 (4)	
C15	0.31559 (19)	0.03344 (19)	0.86501 (11)	0.0351 (4)	
C16	0.1756 (2)	0.0616 (2)	0.94426 (12)	0.0425 (4)	
H16A	0.1724	−0.0396	0.9793	0.051*	
H16B	0.1976	0.1088	0.9854	0.051*	
C17	0.0135 (2)	0.3002 (2)	0.84581 (12)	0.0435 (4)	
C18	−0.1443 (2)	0.4159 (3)	0.82910 (15)	0.0598 (6)	
H18	−0.2374	0.4008	0.8635	0.072*	
C19	−0.1631 (3)	0.5517 (3)	0.76227 (16)	0.0664 (6)	
H19	−0.2689	0.6287	0.7509	0.080*	
C20	−0.0261 (3)	0.5744 (2)	0.71199 (15)	0.0616 (6)	
H20	−0.0388	0.6670	0.6665	0.074*	
C21	0.1304 (2)	0.4599 (2)	0.72885 (13)	0.0488 (5)	
H21	0.2226	0.4767	0.6944	0.059*	
C22	0.1540 (2)	0.3200 (2)	0.79597 (12)	0.0388 (4)	
C23	0.3274 (2)	0.19451 (19)	0.81404 (11)	0.0365 (4)	
H23	0.3724	0.2263	0.8540	0.044*	
C24	0.4482 (2)	0.1651 (2)	0.72960 (12)	0.0386 (4)	
C25	0.5545 (2)	0.2371 (2)	0.67479 (14)	0.0475 (5)	
C26	0.5733 (3)	0.3799 (3)	0.68336 (19)	0.0751 (7)	
H26A	0.6859	0.3688	0.6632	0.113*	
H26B	0.5462	0.3883	0.7467	0.113*	
H26C	0.4999	0.4741	0.6457	0.113*	
C27	0.4710 (2)	−0.0739 (2)	0.90646 (12)	0.0402 (4)	
C28	−0.0485 (2)	−0.0383 (3)	0.79348 (15)	0.0606 (6)	
H28A	−0.1447	−0.0620	0.8035	0.091*	
H28B	−0.0153	−0.0130	0.7284	0.091*	
H28C	−0.0746	0.0510	0.8190	0.091*	
N1	0.64918 (19)	0.16067 (19)	0.61013 (11)	0.0513 (4)	
N2	0.60710 (17)	0.03354 (17)	0.62256 (10)	0.0422 (4)	
N3	0.5899 (2)	−0.1541 (2)	0.94037 (13)	0.0589 (5)	
O1	0.43131 (14)	−0.08065 (13)	0.72675 (8)	0.0395 (3)	
O2	0.01940 (14)	0.16371 (16)	0.91092 (9)	0.0495 (3)	

### Atomic displacement parameters (Å^2^)

**Table:**

	*U*^11^	*U*^22^	*U*^33^	*U*^12^	*U*^13^	*U*^23^
C1	0.0371 (9)	0.0432 (10)	0.0369 (9)	−0.0118 (8)	0.0005 (7)	−0.0076 (8)
C2	0.0514 (12)	0.0729 (15)	0.0632 (14)	−0.0294 (11)	0.0194 (10)	−0.0275 (12)
C3	0.0598 (14)	0.0866 (17)	0.0643 (15)	−0.0265 (13)	0.0232 (11)	−0.0311 (13)
C4	0.0696 (15)	0.0742 (16)	0.0488 (12)	−0.0129 (13)	0.0042 (11)	−0.0275 (11)
C5	0.0709 (15)	0.0600 (13)	0.0532 (12)	−0.0212 (11)	−0.0067 (11)	−0.0209 (10)
C6	0.0444 (11)	0.0536 (12)	0.0445 (11)	−0.0166 (9)	−0.0011 (8)	−0.0117 (9)
C7	0.0306 (8)	0.0361 (9)	0.0403 (9)	−0.0156 (7)	0.0010 (7)	−0.0058 (7)
C8	0.0275 (8)	0.0383 (9)	0.0359 (9)	−0.0132 (7)	0.0016 (7)	−0.0081 (7)
C9	0.0366 (9)	0.0412 (10)	0.0344 (9)	−0.0197 (8)	0.0016 (7)	−0.0109 (7)
C10	0.0400 (10)	0.0418 (10)	0.0521 (11)	−0.0182 (8)	−0.0041 (8)	−0.0106 (8)
C11	0.0577 (12)	0.0386 (10)	0.0553 (12)	−0.0197 (9)	−0.0070 (9)	−0.0086 (9)
C12	0.0688 (14)	0.0495 (12)	0.0483 (11)	−0.0383 (11)	0.0032 (10)	−0.0104 (9)
C13	0.0516 (11)	0.0625 (13)	0.0480 (11)	−0.0372 (11)	0.0039 (9)	−0.0171 (10)
C14	0.0411 (10)	0.0519 (11)	0.0384 (9)	−0.0252 (9)	0.0000 (7)	−0.0128 (8)
C15	0.0301 (8)	0.0373 (9)	0.0364 (9)	−0.0130 (7)	−0.0023 (7)	−0.0081 (7)
C16	0.0382 (10)	0.0479 (10)	0.0391 (10)	−0.0161 (8)	−0.0009 (7)	−0.0108 (8)
C17	0.0371 (10)	0.0483 (11)	0.0419 (10)	−0.0122 (8)	−0.0021 (8)	−0.0154 (8)
C18	0.0355 (10)	0.0707 (15)	0.0603 (13)	−0.0067 (10)	−0.0023 (9)	−0.0221 (11)
C19	0.0480 (13)	0.0658 (15)	0.0627 (14)	0.0032 (11)	−0.0133 (11)	−0.0186 (12)
C20	0.0655 (14)	0.0455 (12)	0.0531 (12)	−0.0026 (10)	−0.0127 (11)	−0.0085 (9)
C21	0.0504 (11)	0.0427 (11)	0.0465 (11)	−0.0125 (9)	−0.0033 (9)	−0.0120 (9)
C22	0.0380 (9)	0.0386 (10)	0.0391 (9)	−0.0115 (8)	−0.0027 (7)	−0.0151 (8)
C23	0.0324 (9)	0.0377 (9)	0.0408 (9)	−0.0142 (8)	−0.0021 (7)	−0.0120 (7)
C24	0.0325 (9)	0.0379 (10)	0.0446 (10)	−0.0153 (8)	0.0011 (7)	−0.0100 (8)
C25	0.0408 (10)	0.0448 (11)	0.0582 (12)	−0.0222 (9)	0.0047 (9)	−0.0129 (9)
C26	0.0767 (16)	0.0669 (15)	0.0984 (19)	−0.0493 (13)	0.0190 (14)	−0.0309 (14)
C27	0.0385 (10)	0.0411 (10)	0.0435 (10)	−0.0179 (9)	−0.0030 (8)	−0.0111 (8)
C28	0.0456 (12)	0.0736 (15)	0.0664 (14)	−0.0299 (11)	−0.0131 (10)	−0.0066 (11)
N1	0.0452 (9)	0.0505 (10)	0.0600 (10)	−0.0278 (8)	0.0125 (8)	−0.0148 (8)
N2	0.0373 (8)	0.0423 (8)	0.0448 (8)	−0.0190 (7)	0.0083 (6)	−0.0114 (7)
N3	0.0509 (10)	0.0516 (10)	0.0744 (12)	−0.0159 (9)	−0.0222 (9)	−0.0108 (9)
O1	0.0375 (6)	0.0400 (7)	0.0420 (7)	−0.0194 (5)	0.0074 (5)	−0.0133 (5)
O2	0.0323 (7)	0.0569 (8)	0.0525 (8)	−0.0161 (6)	0.0036 (6)	−0.0118 (6)

### Geometric parameters (Å, º)

**Table:**

C1—C6	1.367 (3)	C15—C16	1.528 (2)
C1—C2	1.384 (3)	C15—C23	1.550 (2)
C1—N2	1.420 (2)	C16—O2	1.420 (2)
C2—C3	1.372 (3)	C16—H16A	0.9700
C2—H2	0.9300	C16—H16B	0.9700
C3—C4	1.363 (3)	C17—O2	1.377 (2)
C3—H3	0.9300	C17—C22	1.383 (3)
C4—C5	1.374 (3)	C17—C18	1.389 (3)
C4—H4	0.9300	C18—C19	1.364 (3)
C5—C6	1.387 (3)	C18—H18	0.9300
C5—H5	0.9300	C19—C20	1.369 (3)
C6—H6	0.9300	C19—H19	0.9300
C7—N2	1.349 (2)	C20—C21	1.378 (3)
C7—O1	1.354 (2)	C20—H20	0.9300
C7—C24	1.363 (2)	C21—C22	1.388 (3)
C8—O1	1.4550 (19)	C21—H21	0.9300
C8—C9	1.502 (2)	C22—C23	1.520 (2)
C8—C15	1.564 (2)	C23—C24	1.497 (2)
C8—H8	0.9800	C23—H23	0.9800
C9—C10	1.392 (3)	C24—C25	1.406 (2)
C9—C14	1.403 (2)	C25—N1	1.319 (2)
C10—C11	1.375 (3)	C25—C26	1.500 (3)
C10—H10	0.9300	C26—H26A	0.9600
C11—C12	1.377 (3)	C26—H26B	0.9600
C11—H11	0.9300	C26—H26C	0.9600
C12—C13	1.370 (3)	C27—N3	1.139 (2)
C12—H12	0.9300	C28—H28A	0.9600
C13—C14	1.389 (3)	C28—H28B	0.9600
C13—H13	0.9300	C28—H28C	0.9600
C14—C28	1.508 (3)	N1—N2	1.382 (2)
C15—C27	1.472 (2)		
			
C6—C1—C2	120.00 (18)	C15—C16—H16A	109.4
C6—C1—N2	122.32 (16)	O2—C16—H16B	109.4
C2—C1—N2	117.68 (17)	C15—C16—H16B	109.4
C3—C2—C1	120.0 (2)	H16A—C16—H16B	108.0
C3—C2—H2	120.0	O2—C17—C22	123.01 (16)
C1—C2—H2	120.0	O2—C17—C18	115.77 (17)
C4—C3—C2	120.6 (2)	C22—C17—C18	121.14 (19)
C4—C3—H3	119.7	C19—C18—C17	120.1 (2)
C2—C3—H3	119.7	C19—C18—H18	119.9
C3—C4—C5	119.5 (2)	C17—C18—H18	119.9
C3—C4—H4	120.3	C18—C19—C20	120.0 (2)
C5—C4—H4	120.3	C18—C19—H19	120.0
C4—C5—C6	120.7 (2)	C20—C19—H19	120.0
C4—C5—H5	119.7	C19—C20—C21	119.9 (2)
C6—C5—H5	119.7	C19—C20—H20	120.1
C1—C6—C5	119.27 (19)	C21—C20—H20	120.1
C1—C6—H6	120.4	C20—C21—C22	121.67 (19)
C5—C6—H6	120.4	C20—C21—H21	119.2
N2—C7—O1	122.29 (15)	C22—C21—H21	119.2
N2—C7—C24	109.30 (15)	C17—C22—C21	117.23 (17)
O1—C7—C24	128.28 (15)	C17—C22—C23	121.57 (16)
O1—C8—C9	107.74 (13)	C21—C22—C23	121.20 (16)
O1—C8—C15	109.91 (13)	C24—C23—C22	115.21 (14)
C9—C8—C15	114.90 (14)	C24—C23—C15	106.57 (13)
O1—C8—H8	108.0	C22—C23—C15	108.94 (13)
C9—C8—H8	108.0	C24—C23—H23	108.7
C15—C8—H8	108.0	C22—C23—H23	108.7
C10—C9—C14	119.09 (16)	C15—C23—H23	108.7
C10—C9—C8	119.30 (15)	C7—C24—C25	103.93 (15)
C14—C9—C8	121.58 (15)	C7—C24—C23	121.78 (15)
C11—C10—C9	121.61 (17)	C25—C24—C23	134.00 (16)
C11—C10—H10	119.2	N1—C25—C24	111.93 (16)
C9—C10—H10	119.2	N1—C25—C26	119.44 (17)
C10—C11—C12	119.34 (18)	C24—C25—C26	128.61 (18)
C10—C11—H11	120.3	C25—C26—H26A	109.5
C12—C11—H11	120.3	C25—C26—H26B	109.5
C13—C12—C11	119.63 (18)	H26A—C26—H26B	109.5
C13—C12—H12	120.2	C25—C26—H26C	109.5
C11—C12—H12	120.2	H26A—C26—H26C	109.5
C12—C13—C14	122.41 (17)	H26B—C26—H26C	109.5
C12—C13—H13	118.8	N3—C27—C15	177.91 (19)
C14—C13—H13	118.8	C14—C28—H28A	109.5
C13—C14—C9	117.83 (17)	C14—C28—H28B	109.5
C13—C14—C28	118.88 (16)	H28A—C28—H28B	109.5
C9—C14—C28	123.28 (16)	C14—C28—H28C	109.5
C27—C15—C16	107.11 (14)	H28A—C28—H28C	109.5
C27—C15—C23	108.72 (13)	H28B—C28—H28C	109.5
C16—C15—C23	108.72 (14)	C25—N1—N2	105.44 (14)
C27—C15—C8	110.90 (14)	C7—N2—N1	109.38 (14)
C16—C15—C8	112.16 (13)	C7—N2—C1	131.23 (15)
C23—C15—C8	109.13 (14)	N1—N2—C1	119.35 (14)
O2—C16—C15	111.34 (14)	C7—O1—C8	111.93 (12)
O2—C16—H16A	109.4	C17—O2—C16	116.17 (13)
			
C6—C1—C2—C3	−1.4 (3)	C20—C21—C22—C23	−179.94 (17)
N2—C1—C2—C3	179.1 (2)	C17—C22—C23—C24	139.64 (17)
C1—C2—C3—C4	0.8 (4)	C21—C22—C23—C24	−40.7 (2)
C2—C3—C4—C5	0.4 (4)	C17—C22—C23—C15	20.0 (2)
C3—C4—C5—C6	−1.0 (4)	C21—C22—C23—C15	−160.35 (15)
C2—C1—C6—C5	0.9 (3)	C27—C15—C23—C24	71.73 (17)
N2—C1—C6—C5	−179.67 (17)	C16—C15—C23—C24	−171.97 (13)
C4—C5—C6—C1	0.3 (3)	C8—C15—C23—C24	−49.35 (16)
O1—C8—C9—C10	54.68 (19)	C27—C15—C23—C22	−163.39 (13)
C15—C8—C9—C10	−68.2 (2)	C16—C15—C23—C22	−47.10 (17)
O1—C8—C9—C14	−127.45 (16)	C8—C15—C23—C22	75.53 (16)
C15—C8—C9—C14	109.68 (17)	N2—C7—C24—C25	−1.34 (19)
C14—C9—C10—C11	−2.2 (3)	O1—C7—C24—C25	174.53 (17)
C8—C9—C10—C11	175.76 (16)	N2—C7—C24—C23	−175.98 (15)
C9—C10—C11—C12	−0.6 (3)	O1—C7—C24—C23	−0.1 (3)
C10—C11—C12—C13	2.6 (3)	C22—C23—C24—C7	−101.70 (19)
C11—C12—C13—C14	−1.7 (3)	C15—C23—C24—C7	19.3 (2)
C12—C13—C14—C9	−1.1 (3)	C22—C23—C24—C25	85.5 (2)
C12—C13—C14—C28	177.50 (18)	C15—C23—C24—C25	−153.51 (19)
C10—C9—C14—C13	3.0 (2)	C7—C24—C25—N1	1.3 (2)
C8—C9—C14—C13	−174.90 (15)	C23—C24—C25—N1	174.98 (18)
C10—C9—C14—C28	−175.56 (17)	C7—C24—C25—C26	−176.9 (2)
C8—C9—C14—C28	6.6 (3)	C23—C24—C25—C26	−3.3 (4)
O1—C8—C15—C27	−53.68 (18)	C24—C25—N1—N2	−0.8 (2)
C9—C8—C15—C27	68.02 (18)	C26—C25—N1—N2	177.66 (18)
O1—C8—C15—C16	−173.39 (13)	O1—C7—N2—N1	−175.21 (15)
C9—C8—C15—C16	−51.69 (19)	C24—C7—N2—N1	0.96 (19)
O1—C8—C15—C23	66.07 (16)	O1—C7—N2—C1	2.1 (3)
C9—C8—C15—C23	−172.23 (13)	C24—C7—N2—C1	178.30 (17)
C27—C15—C16—O2	−179.60 (14)	C25—N1—N2—C7	−0.1 (2)
C23—C15—C16—O2	63.08 (17)	C25—N1—N2—C1	−177.82 (16)
C8—C15—C16—O2	−57.70 (19)	C6—C1—N2—C7	18.8 (3)
O2—C17—C18—C19	−176.47 (18)	C2—C1—N2—C7	−161.74 (19)
C22—C17—C18—C19	0.3 (3)	C6—C1—N2—N1	−164.07 (17)
C17—C18—C19—C20	−0.4 (3)	C2—C1—N2—N1	15.4 (3)
C18—C19—C20—C21	0.2 (3)	N2—C7—O1—C8	−170.88 (15)
C19—C20—C21—C22	0.2 (3)	C24—C7—O1—C8	13.7 (2)
O2—C17—C22—C21	176.57 (15)	C9—C8—O1—C7	−171.15 (13)
C18—C17—C22—C21	0.0 (3)	C15—C8—O1—C7	−45.26 (17)
O2—C17—C22—C23	−3.8 (3)	C22—C17—O2—C16	17.4 (2)
C18—C17—C22—C23	179.67 (16)	C18—C17—O2—C16	−165.84 (16)
C20—C21—C22—C17	−0.3 (3)	C15—C16—O2—C17	−47.30 (19)

### Hydrogen-bond geometry (Å, º)

Cg1 is the centroid of the C1–C6 ring.

**Table:**

*D*—H···*A*	*D*—H	H···*A*	*D*···*A*	*D*—H···*A*
C2—H2···N1	0.93	2.42	2.759 (3)	102
C6—H6···O1	0.93	2.37	2.952 (2)	120
C28—H28*C*···O2	0.96	2.47	3.310 (3)	146
C16—H16*A*···O2^i^	0.97	2.56	3.380 (2)	143
C20—H20···*Cg*1^ii^	0.96	2.86	3.567	134

Symmetry codes: (i) −*x*, −*y*, −*z*+2; (ii) *x*−1, *y*+1, *z*.
